# Metabolic effects of estradiol versus testosterone in complete androgen insensitivity syndrome

**DOI:** 10.1007/s12020-022-03017-8

**Published:** 2022-03-08

**Authors:** Matthias K. Auer, Wiebke Birnbaum, Michaela F. Hartmann, Paul-Martin Holterhus, Alexandra Kulle, Anke Lux, Luise Marshall, Katarina Rall, Annette Richter-Unruh, Ralf Werner, Stefan A. Wudy, Olaf Hiort

**Affiliations:** 1 Medizinische Klinik and Poliklinik IV, Klinikum der Universität München, LMU München, Munich, Germany; 2 Division of Pediatric Endocrinology and Diabetes, Department of Pediatric and Adolescent Medicine, University of Lübeck, Lübeck, Germany; 3 Steroid Research & Mass Spectrometry Unit, Laboratory for Translational Hormone Analytics in Pediatric Endocrinology, Division of Pediatric Endocrinology & Diabetology, Centre of Child and Adolescent Medicine, Justus Liebig University, Giessen, Germany; 4 Division of Pediatric Endocrinology and Diabetes, Department of Pediatrics, University Hospital of Schleswig – Holstein, Campus Kiel/Christian – Albrechts University of Kiel, Kiel, Germany; 5 Institute for Biometrics and Medical Informatics, Otto-von-Guericke University, Magdeburg, Germany; 6 Department of Women’s Health, Centre for Rare Female Genital Malformations, Women’s University Hospital, Tübingen University Hospital, Tübingen, Germany; 7 Paediatric Endocrinology, Department of Pediatrics, Universitätsklinikum Münster, Westfälische Wilhelms-Universität Münster, Münster, Germany; 8 Institute for Molecular Medicine, University of Lübeck, Lübeck, Germany

**Keywords:** CAIS, Androgen receptor, Estradiol, Testosterone, Metabolism

## Abstract

**Purpose:**

To study differences in metabolic outcomes between testosterone and estradiol replacement in probands with complete androgen insensitivity syndrome (CAIS).

**Methods:**

In this multicentre, double-blind, randomized crossover trial, 26 women with CAIS were included of whom 17 completed the study. After a two-months run in phase with estradiol, probands either received transdermal estradiol followed by crossover to transdermal testosterone or vice versa. After six months, differences in lipids, fasting glucose, insulin, hematocrit, liver parameters and blood pressure between the treatment phases were investigated.

**Results:**

Linear mixed models adjusted for period and sequence did not reveal major group differences according to treatment for the investigated outcomes. In each treatment group, there were however significant uniform changes in BMI and cholesterol. BMI increased significantly, following six months of estradiol ( + 2.7%; *p* = 0.036) as well as testosterone treatment ( + 2.8%; *p* = 0.036). There was also a significant increase in total ( + 10.4%; *p* = 0.001) and LDL-cholesterol ( + 29.2%; *p* = 0.049) and a decrease in HDL-cholesterol (−15.8%; *p* < 0.001) following six months of estradiol as well as six months of testosterone treatment (total cholesterol: + 14.6%; *p* = 0.008; LDL-cholesterol: + 39.1%; *p* = 0.005, HDL-cholesterol: −15.8%; *p* = 0.004). Other parameters remained unchanged.

**Conclusion:**

Transdermal estradiol as well as testosterone treatment in women with CAIS results in worsening in lipid profiles. Given the relatively small sample size, subtle group differences in other metabolic parameters may have remained undetected.

## Introduction

Complete androgen insensitivity syndrome (CAIS) is the most common 46, XY disorder of sex development (DSD) with an estimated prevalence of 1 in 20. 000–90. 000 births or 4·1:100 000 girls [1]. It is characterized by complete loss of androgen receptor functioning due to X-linked recessive mutations within the androgen receptor (AR) gene [2] and subsequent development of a complete external female phenotype. Subjects lack Müllerian duct structures and androgen-dependent body hair.

The endocrine profile after puberty is usually characterized by testosterone concentrations in the usual to upper male reference range, while estradiol concentrations are normal to slightly increased relative to normal male references originating primarily from testicular secretion and peripheral aromatization of androstenedione and testosterone [3]. However, despite aromatization, estradiol concentrations are usually below the usual female reference range [4]. Due to the unknown risk for developing gonadal tumors, women with CAIS usually underwent early gonadectomy until recently [2] and were then depended on sex hormone replacement therapy. So far, the treatment of patients with CAIS after gonadectomy has basically followed the established concepts for the therapy of female hypogonadism. However, this results in the replacement of previously high endogenous androgen concentrations by estrogens [2].

Here we report the results of a secondary metabolic outcome analysis of the first multicentre, randomised, double-dummy, double-blind crossover trial investigating the effects of estradiol in comparison to testosterone replacement therapy in CAIS probands [5]. We could show that androgen replacement seems to be non-inferior to estradiol in terms of quality of life and does result in comparable levels of estrogens. In addition, we could demonstrate that testosterone treatment may have beneficial effects on sexual functioning in CAIS. Although it may seem unlikely that testosterone should exert any distinct effect from that of estradiol in these probands at first glance, hypothetical considerations have underscored the anecdotally reported improvements in general well-being following androgen replacement [6]. With regard to general wellbeing and sexual functioning it has to be kept in mind that testosterone is not only metabolized to estradiol but especially in terms of so-called neurosteroids [7] may also be converted to metabolites that exert their activity neither via estrogen - nor androgen receptors. Furthermore, differences in treatment effects might be plausible assuming that there is a difference between a systemic increase in estradiol in comparison to a local increase depending on the distribution of aromatase expression in the corresponding target tissue [8]. Hence, local estradiol concentrations can significantly differ independent of systemic levels.

Little is known about the metabolic features of CAIS women even though androgens are known to modulate a wide range of cardiometabolic parameters in both sexes [9]. There is evidence that e.g., lack of androgen activity in these probands may have a negative effect on bone mineral density, due to the distinct effects of testosterone and estradiol on bone metabolism [10]. However, most effects on bone by testosterone seem to be primarily mediated by local conversion into estradiol via aromatase enzyme activity [11].

A small study indicated a less favorable cardiometabolic profile in CAIS women in comparison to control women (with gonadal dysgenesis), including lower fat free mass and elevated total and LDL-cholesterol as well as an increase in insulin resistance [12].

We hypothesized that there would be no significant differences in terms of metabolic parameters between CAIS probands receiving testosterone versus estradiol replacement therapy namely, BMI, total cholesterol, LDL-cholesterol, HDL-cholesterol, triglyceride and fasting glucose and insulin-levels.

## Subjects and methods

### Study design and participants

The complete study design has been reported elsewhere [5]. In summary the study has been performed at three university medical centers and three specialized treatment institutions in Germany (Lübeck, Berlin, Regensburg, Tübingen, Bochum [Dortmund], and Munich) between November 2011 and January 2016. Diagnosis of CAIS was confirmed by molecular genetic analysis of the AR gene. Gonadectomy had to date back more than 1 year before inclusion in the study and the timing of gonadectomy varied considerably individually in the cohort between prepubertal and postpubertal [5]. Those participants who were gonadectomized late entered puberty as typical for CAIS.

Exclusion criteria were:

•Disorder of Sex Development other than complete androgen insensitivity syndrome

•Steroid medication other than study trial medication

•Gonads in situ

•Disorder of liver function

•Chronic skin disease

•Serious chronic disorders affected by sex steroid medication

•Malignant disorders

•Severe psychiatric disorders

**Fig. 1:**
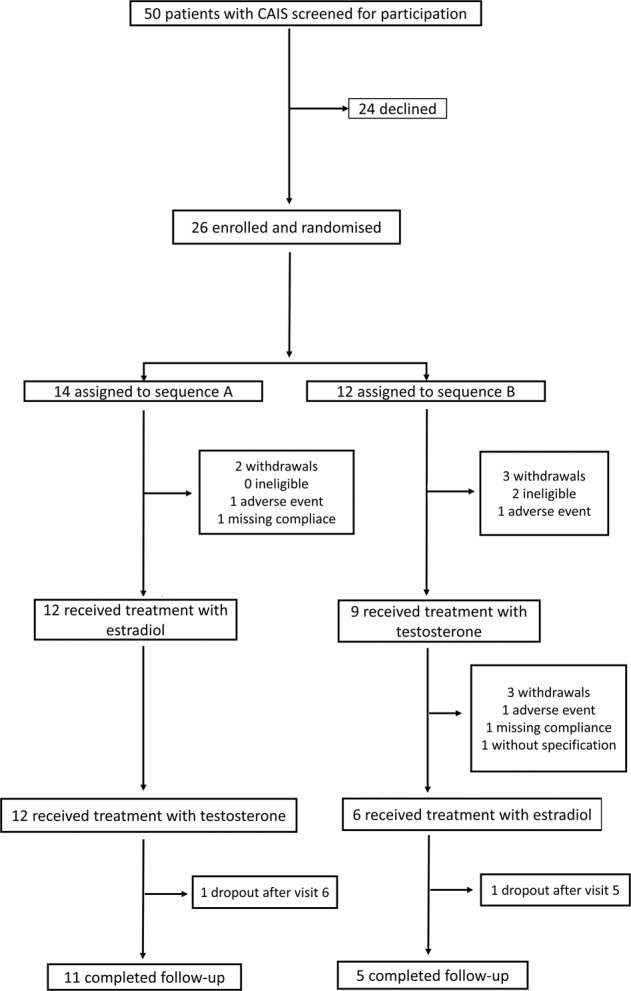
Trial profile

26 women with CAIS aged 18–54 years were included. Secondary analyses included the per-protocol population. Ten probands left the study before completion. Eight left during the run-in, respectively treatment phase. One proband did not attend visit six. As per protocol, data from visit five was used in this case. One proband did not attend the final follow-up visit and was included in the final analysis as well. Two probands were incompliant and had to be excluded. Finally, 12 probands in sequence A and six probands in sequence B were included in our analysis (Fig. 1). There were neither differences in demographic, anthropometric nor in medical variables between subjects who completed the study to those who did not (10.6084/m9.figshare.16944979.v).

### Treatment

All participants received standard estradiol 1.5 mg/day during a 2-month run-in phase to accomplish a homogeneous hormonal milieu, as from the initial cohort five probands were not receiving any sex hormone treatment at study inclusion and in one proband it was unclear, if hormone replacement in the preceding month had taken place. The final cohort included four probands without recent hormone replacement. This approach instead of a wash-out period was chosen to avoid leaving the participants without any hormonal replacement, resulting in a completely unphysiological state at the start of the trial.

**Fig. 2:**
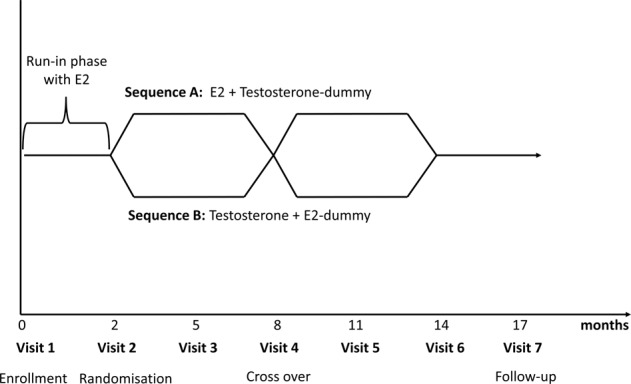
Study design. All participants received standard estradiol 1.5 mg/day during a 2-month run-in phase to accomplish a homogeneous hormonal milieu. Participants were randomly assigned (14:12) to receive estradiol 1.5 mg/day and a testosterone dummy for 6 months followed by crossover to testosterone 50 mg/day and estradiol dummy for 6 months (sequence A) or to receive testosterone 50 mg/day and estradiol dummy for 6 months followed by crossover to estradiol 1.5 mg/day and testosterone dummy for 6 months (sequence B). The crossover of active component after 6 months was done in a double-blind fashion

Participants were randomly assigned (14:12) to receive estradiol (Gynokadin©; Dr Kade Pharmaceuticals, Berlin, Germany) 1.5 mg/day ( = 2.5 g gel) and a testosterone dummy for 6 months followed by crossover to testosterone (Testogel©; BESINS Healthcare SA, Brussels, Belgium) 50 mg/day ( = 5 g gel) and estradiol dummy for 6 months (sequence A) or to receive testosterone 50 mg/day and estradiol dummy for 6 months followed by crossover to estradiol 1.5 mg/day and testosterone dummy for 6 months (sequence B; Fig. 2). The crossover of active component after 6 months was done in a double-blinded manner. The dummy for each study drug was provided by BESINS and Dr. Kade Pharmaceuticals. Bioavailability for the testosterone gel is estimated to lie between 9 and 15% [13] and for the estradiol gel around 6% [14].

### Hormonal analysis

Testosterone and estradiol were measured by Liquid-chromatography - tandem mass spectrometry (LC-MS/MS) at the Division of Pediatric Endocrinology and Diabetes, Department of Pediatrics, University Hospital of Schleswig – Holstein, Campus Kiel, Christian-Albrechts University of Kiel with the facilities provided by a previous BMBF-funding [15, 16]. Luteinizing hormone (LH) and follicle-stimulating hormone (FSH) were measured in Lübeck by the Roche Elecsys©. The reproducibility for testosterone was lower than 7.1%, reported in Table 2 [15]. For estradiol it was lower than 5% [17]. Four times a year a round-robin test was performed for both hormones at the Reference Institute for Bioanalysis.

### Laboratory measurements

Cholesterol [total, HDL, LDL], triglycerides, insulin, as well as safety parameters γGT (gamma-glutamyltransferase), GOT (glutamic oxaloacetic transaminase), GPT (alanine transaminase), AP (alcalic phosphatase) were determined centrally in the laboratories of the University-Hospital Schleswig-Holstein, Campus Lübeck. Haematocrit and haemoglobin were analysed locally at the study sites due to instability of the samples. Due to sampling processing issues at one centre 4–5 samples had to be excluded from analysis of AP, fasting glucose and insulin levels. Pathological levels for cholesterol were defined as follows: total cholesterol > 4.9 mmol/l; LDL-cholesterol > 3.0 mmol/l; HDL-cholesterol < 1.2 mmol/l.

### Statistical analysis

To compensate for unbalanced dropout between the treatment groups, differences between the two treatments for the secondary endpoints were tested in a mixed linear model analysis for crossover designs, with fixed effects for treatment (estradiol vs testosterone), period (first vs second treatment phase), and sequence (estradiol to testosterone vs testosterone to estradiol) and with a random patient effect. The analysis was based on data at the end of the two treatment phases (visits 4 and 6, respectively). Laboratory parameters were log-transformed to approximate a Gaussian distribution if necessary. In a further exploratory secondary analysis, the Wilcoxon paired difference test was used to compare data from estradiol or testosterone treatments (independent of sequence or period) against the respective baseline values and to compare data from estradiol or testosterone treatments. To exclude the possibility that changes in the laboratory parameters were only due to changes in the BMI, corresponding correlation analyses and analyses of covariance were performed considering the difference values of the BMI. IBM SPSS Statistics version 24 was used for statistical analyses. A *p*-value less than 0·05 was used to indicate statistical significance.

## Results

Results of hormone measures have been reported before [5]. In brief, after run-in treatment with estradiol, median estradiol concentrations were 170 pmol/l and thus within the lower reference range for women and remained stable during estradiol treatment. Median estradiol concentrations during testosterone treatment phase were 100 pmol/l. Median testosterone concentrations during the run-in phase (0.63 nmol/l) and during estradiol treatment (90 pmol/l) were in the lower range for adult women. The median testosterone concentration during testosterone treatment (15.6 nmol/l) was within the range for young adult men.

**Fig. 3:**
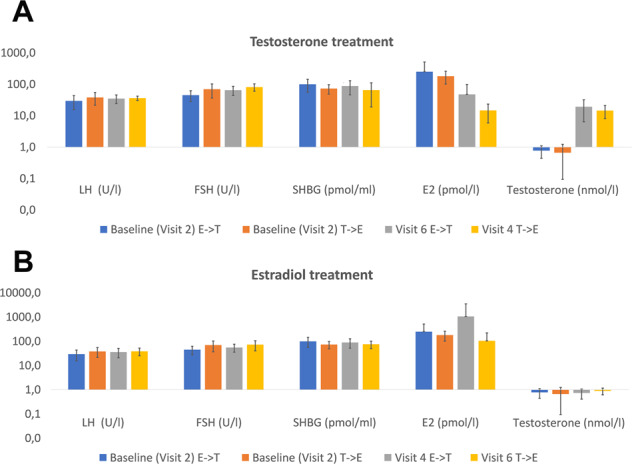
Changes in hormonal parameters separated by sequence. Changes in hormonal parameters under estradiol (**A**) and testosterone (**B**) treatment. Y-axis is log-transformed. E Estradiol, T Testosterone, -> Sequence. Means and SD are depicted

The concentrations of LH and FSH hormone were high before treatment (After run-in: LH 33.9IU/l; FSH 55.7IU/l) and remained high after treatment. No significant difference was found in gonadotrophin concentrations between treatment sequences. Changes according to sequence are presented in Fig. 3. There was no significant difference at baseline regarding metabolic outcome variables as depicted in Table 1.

**Table 1:** Baseline characteristics of the whole study sample at baseline

	*N*	Mean	SD	p*
Age (years)	26	32.7	9.5	0.133
Age at gonadectomy (years)	26	17.2	8.1	0.153
Weight (kg)	26	74.7	12.4	0.950
BMI (kg/m²)	26	25.1	4.0	0.860
Systolic blood pressure (mmHg)	26	120.7	14.4	0.950
Diastolic blood pressure (mmHg)	26	76.1	10.3	0.568
Total cholesterol (mmol/l)	23	4.9	1.0	0.255
HDL-cholesterol (mmol/l)	24	1.8	0.5	0.395
LDL-cholesterol (mmol/l)	24	2.5	1.1	0.989
Triglycerides (mmol/l)	24	1.0	0.5	1.000
Glucose (mmol/l)	24	5.0	1.5	0.635
Insulin (mlU/l)	23	11.6	9.4	0.335
HOMA-IR	23	2.7	2.6	0.228
Hemoglobin (g/dl)	25	13.7	0.7	0.187
Hematocrit (%)	25	40.5	2.1	0.086
GOT (U/l)	24	20.8	6.0	0.897
GPT (U/l)	24	15.4	6.9	0.157
GGT (U/l)	24	14.1	5.8	0.479
Alkaline phosphatase (U/L)	24	62.4	14.9	0.720
LH (IU/l)	25	34.0	15.8	0.979
FSH (IU/l)	25	62.5	31.0	0.687
SHBG (pmol/ml)	25	100.7	50.4	0.434
Estradiol Kiel (pmol/l)	24	89.4	119.9	0.277
Testosteron (Kiel) (nmol/l)	24	0.6	0.4	0.910

^*^Differences between groups stratified by sequence (Mann-Whitney-Test)

With the exception of AP-levels, which were significantly lower in the estradiol (60 U/l; 95%CI 52.9–67.2) than in the testosterone group (64.4 U/l; 95%CI 57.4–71.4; *p* = 0.046) no significant differences were found in the effect of estradiol and testosterone on any other of the investigated parameters in the linear mixed model (Table 2). There were no significant changes in the investigate parameters during the 2 months run-in phase (data not shown).

**Table 2:** Treatment effect, data derived from linear mixed model analysis

	Treatment	Mean	95% CI		*p*-value	*p*-value period	*p*-value sequence
			Lower	Upper			
Weight (kg)	Oestradiol	74.78	66.73	82.38	0.126	0.354	0.421
	Testosterone	75.72	67.68	83.76			
	Difference	−0.94	−2.15	0.27			
BMI (kg/m²)	Oestradiol	24.66	22.39	26.93	0.132	0.330	0.435
	Testosterone	24.95	22.69	27.21			
	Difference	−0.29	−0.67	0.09			
Systolic blood pressure (mmHg)	Oestradiol	120.15	114.45	125.86	0.442	0.847	0.210
	Testosterone	121.96	116.46	127.46			
	Difference	−1.81	−6.49	2.87			
Diastolic blood pressure (mmHg)	Oestradiol	75.48	72.01	78.96	0.724	0.754	**0.012**
	Testosterone	74.91	71.59	78.24			
	Difference	0.57	−2.63	3.76			
Total cholesterol* (mmol/l)	Oestradiol	5.33	4.89	5.77	0.260	**0.006**	0.423
	Testosterone	5.50	5.09	5.92			
	Difference	−0.17	−0.49	0.14			
HDL-cholesterol* (mmol/l)	Oestradiol	1.68	1.47	1.89	0.915	**0.001**	0.093
	Testosterone	1.71	1.51	1.90			
	Difference	−0.03	−0.19	0.12			
LDL-cholesterol* (mmol/l)	Oestradiol	3.01	2.44	3.58	0.221	**0.001**	0.936
	Testosterone	3.13	2.58	3.68			
	Difference	−0.12	−0.48	0.23			
Triglycerides* (mmol/l)	Oestradiol	0.77	0.62	0.92	0.638	0.730	0.532
	Testosterone	0.78	0.63	0.92			
	Difference	−0.004	−0.11	0.10			
Glucose (mmol/l)	Oestradiol	4.86	4.28	5.45	0.434	0.702	0.313
	Testosterone	5.02	4.45	5.58			
	Difference	−0.15	−0.54	0.24			
Insulin (mlU/l)	Oestradiol	5.64	3.73	7.55	0.416	0.828	0.731
	Testosterone	6.19	4.35	8.04			
	Difference	−0.55	−1.89	0.80			
HOMA-IR	Oestradiol	1.75	0.50	2.99	0.578	0.448	0.310
	Testosterone	1.35	0.18	2.52			
	Difference	0.40	0.32	0.47			
Hematocrit* (%)	Oestradiol	39.92	38.64	41.19	0.538	0.263	0.464
	Testosterone	40.13	38.89	41.38			
	Difference	−0.21	−0.96	0.53			
Hemoglobin* (g/dl)	Oestradiol	13.65	13.21	14.10	0.752	0.214	0.332
	Testosterone	13.61	13.18	14.04			
	Difference	0.05	−0.24	0.33			
GOT* (U/l)	Oestradiol	21.56	19.07	24.04	0.144	0.543	0.143
	Testosterone	23.33	21.06	25.60			
	Difference	−1.77	−4.21	0.66			
GPT* (U/l)	Oestradiol	17.17	14.24	20.11	0.858	0.889	0.224
	Testosterone	17.12	14.44	19.82			
	Difference	0.05	−2.74	2.84			
GGT* (U/l)	Oestradiol	15.01	12.04	17.97	0.641	0.263	0.695
	Testosterone	15.12	12.30	17.94			
	Difference	−0.11	−2.19	1.96			
AP* (U/l)	Oestradiol	60.04	52.88	67.20	**0.046**	0.999	0.666
	Testosterone	64.36	57.36	71.36			
	Difference	−4.33	−8.96	0.31			

Bold values indicates statistically significant *p* values

^*^Log transformed

There was a significant increase in BMI following six months of estradiol ( + 2.7%, *z* = 2.107; *p* = 0.036) as well as testosterone treatment ( + 2.8%, *z* = −2.101; *p* = 0.036) in comparison to visit 2 after the run-in phase. There was also a significant increase in total ( + 10.4%, *z* = −3.409; *p* = 0.001) and LDL-cholesterol ( + 29.2%, *z* = 3.510; *p* < 0.001) and a decrease in HDL-cholesterol (−15.8%, *z* = −1.965; *p* < 0.049) during six months of treatment with estradiol (Table 3). A similar pattern was seen following the testosterone sequence (total cholesterol: + 14.6%, *z* = −2.636; *p* = 0.008; LDL-cholesterol: + 39.1%, *z* = −2.832; *p* = 0.005, HDL-cholesterol: −15.8%, *z* = −2.912; *p* = 0.004) (Table 4). There was no correlation between the changes in lipid levels and those in the BMI and no effect of BMI in the ANCOVA, suggesting that the differences in lipid levels were treatment specific (data not shown).

**Table 3:** Treatment with Oestradiol

	Visit 2			Visit 4/6				
	*N*	Mean	SD	*N*	Mean	SD	%	p*
Weight (kg)	17	74.4	15.7	17	76.5	18.4	2.9	**0.035**
BMI (kg/m²)	17	24.6	4.4	17	25.3	5.1	2.7	**0.036**
Systolic blood pressure (mmHg)	17	122.9	14.0	18	120.3	13.4		0.381
Diastolic blood pressure (mmHg)	16	74.8	9.9	17	76.8	8.6		0.569
Total cholesterol (mmol/l)	17	4.8	0.9	17	5.3	1.0	11.4	**0.001**
HDL-Cholesterol (mmol/l)	17	1.9	0.4	17	1.6	0.5	−13.0	**0.049**
LDL-Cholesterol (mmol/l)	17	2.4	0.9	17	3.1	1.2	28.1	**<0.001**
Triglycerides (mmol/l)	17	0.7	0.3	17	0.9	0.4		0.184
Glucose (mmol/l)	14	5.1	0.7	14	5.0	1.0		0.900
Insulin (mlU/l)	12	4.4	3.2	12	5.7	4.3		0.477
HOMA-IR	12	2.2	3.5	12	2.5	5.4		0.804
Hemoglobin (g/dl)	14	13.7	0.9	15	13.6	1.0		0.916
Hematocrit (%)	13	40.2	2.7	14	39.8	3.2		0.686
GOT (U/l)	17	23.2	7.9	17	21.9	4.4		0.798
GPT (U/l)	17	16.9	5.4	17	17.2	7.5		0.887
GGT (U/l)	17	14.6	4.5	17	16.3	9.2		0.328
Alkaline phosphatase (U/L)	13	63.6	15.7	14	62.7	18.0		0.780

Bold values indicates statistically significant *p* values

*Between visit 2 and 4/6

**Table 4:** Treatment with Testosterone

		Visit 2		Visit 4/6				
	*N*	Mean	SD	*N*	Mean	SD	%	p*
Weight (kg)	18	73.9	15.4	18	75.8	17.0	2.6	0.056
BMI (kg/m²)	18	24.4	4.4	18	25.0	4.7	2.4	**0.036**
Systolic blood pressure (mmHg)	17	122.9	14.0	18	120.3	13.4		0.381
Diastolic blood pressure (mmHg)	16	74.9	10.0	17	76.9	9.3		0.477
Total cholesterol (mmol/l)	18	4.8	0.9	18	5.5	1.0	14.9	**0.008**
HDL-Cholesterol (mmol/l)	17	1.9	0.4	17	1.6	0.6	−16.2	**0.004**
LDL-Cholesterol (mmol/l)	18	2.3	0.9	18	3.2	1.4	38.7	**0.005**
Triglycerides (mmol/l)	18	0.7	0.3	18	0.8	0.4	15.4	0.459
Glucose (mmol/l)	14	5.1	0.7	14	5.4	0.9		0.173
Insulin (mlU/l)	13	5.4	4.7	14	6.2	4.8		0.184
HOMA-IR	13	1.2	1.2	14	1.5	1.3		0.151
Hemoglobin (g/dl)	16	13.8	1.0	17	13.8	0.8		0.864
Hematocrit (%)	16	40.8	2.6	17	40.6	2.4		0.894
GOT (U/l)	18	23.0	7.7	18	24.2	7.5		0.538
GPT (U/l)	18	16.6	5.4	18	16.8	5.8		0.931
GGT (U/l)	18	14.7	4.4	18	15.0	5.9		0.593
Alkaline phosphatase (U/L)	14	62.5	15.3	63.5	45.0	106.0		0.084

Bold values indicates statistically significant *p* values

*Between visit 2 and 4/6

At visit two, nine probands (52.9%) in the estradiol arm had pathologically elevated total cholesterol levels. Three probands with initially normal cholesterol levels developed pathological levels following six months of E2 treatment. Before randomization, all probands had HDL-cholesterol levels within the normal range of whom four developed pathologically low levels during treatment. Regarding LDL-cholesterol, from twelve probands (70.6%) with initially normal levels at visit two, five developed pathological levels during treatment.

In the testosterone arm, nine probands (50%) had normal cholesterol levels, while the other half had pathologically elevated cholesterol levels. Among these, following six months of T treatment, four developed elevated levels. Total cholesterol normalized in one proband with initially elevated levels. HDL-cholesterol levels were normal in all probands at visit two. Among these, in five decreased levels into the pathological range during treatment. LDL-cholesterol was normal in 13 probands and elevated in five probands. In four probands, LDL-cholesterol levels rose into the pathological range while in one proband LDL-cholesterol normalized in the course of treatment.

There were no differences in blood pressure, hemoglobin/hematocrit, triglycerides, liver parameters or insulin/glucose following any treatment sequence. There was no significant change in either parameter between visit 1 and 2 ( = run-in-phase, data not shown).

## Discussion

This is the first study investigating two different treatment options in CAIS individuals with regard to metabolic effects in a randomised controlled fashion. Both treatments resulted in a less favorable lipid profile, as there was a significant increase in total and LDL-cholesterol and a significant decrease in HDL-cholesterol. In addition, there was a slight but significant increase in BMI in both groups. Changes in cholesterol levels were however independent of changes in weight. Although the observed changes may be due to a simple time effect this is unlikely in view of the relatively short time period of six months in each treatment phase. Changes might be clinically significant as in almost half of probands with initially normal LDL-cholesterol levels in both treatment arms, levels rose into the pathological range. There were no significant differences between both treatments in terms of metabolic and safety parameters. Although the study might be underpowered regarding the detection of more subtle changes in the investigated outcomes, the results do not indicate that there are major differences between both treatments.

Despite the fact that there was no significant change in lipid parameters in our study within the 2-months run-in phase, it might have played a role that not all probands had received hormonal treatment in the last months before inclusion in the study and also in those who did, steroid exposure might have been different from that provided in a controlled fashion during participation in the trial where medication use was well monitored.

As being evident by the sexually dimorphic fat distribution pattern emerging with onset of puberty [18], sex steroids and in particular estradiol have significant implications in fat and lipid metabolism and regulation [19]. Ovariectomy in rodent models, as well as natural menopause in women, results in increases in adipose tissue [20] preferably in the abdominal region. These changes can be reversed by estrogen replacement [21, 22]. Also, in men, aromatization of testosterone to estradiol seems to be crucial in terms of energy homeostasis and lipid distribution [23]. Men with aromatase deficiency [24] as well as estrogen receptor defects [25] e.g., present with features of the metabolic syndrome despite normal testosterone levels. In men with aromatase deficiency estradiol substitution results in a significant increase in HDL- and decrease in LDL- cholesterol [24].

That estradiol, as well as testosterone treatment in our study, resulted in worsening of lipid parameters was unexpected and resemble the effects seen in hyperandrogenism in women [26]. There is further evidence that there might be a U-shaped relationship between androgen levels and cardiometabolic risk in women [27]. However, this is not a uniform finding [28] and it cannot conclusively explain the detrimental effects of both treatments in our study.

High concentrations of testosterone e.g., as seen in gender-affirming hormone treatment (GAHT) in transgender men ( = male gender identity) result in a decrease in subcutaneous fat with unchanged visceral fat depots [9, 29]. In addition, GAHT usually results in an unfavorable lipid profile with a decrease in HDL- and an increase in LDL-cholesterol [9]. In contrast, aside from CAIS, a clear human model for isolated hypoandrogenism without estradiol deficiency is missing. We can therefore only speculate on the cause of the changes observed in our study.

Unfortunately, there is a paucity of studies on metabolic characteristic in women with CAIS in general and on the effects of hormone replacement in particular that could help us to classify our results. An unfavorable metabolic profile in women with CAIS receiving estrogen replacement was also documented in a small cross-sectional study by Dati and colleagues [12]. CAIS women presented with lower fat free mass and elevated total and LDL-cholesterol levels and increased insulin resistance in comparison to control women. While overall mean BMI was not significantly increased, prevalence of obesity was higher in women with CAIS than expected for the corresponding Italian reference population. The comparability with our study is however limited by the fact that probands in the aforementioned study received various regimens of estrogen replacement and 23% of probands included had not undergone gonadectomy and therefore preserved testosterone secretion.

In another study by Tsimaris and colleagues [30] the effects of six months sex hormone replacement on cardiometabolic parameters were investigated in 23 probands with 46, XY DSD. Hormone therapy was initiated either after gonadectomy, or after a 3-months wash-out period of prior HT. In contrast to our study, the authors could show that hormone replacement therapy resulted in a raise in HDL cholesterol and a decrease in triglyceride levels, while there were no changes in total and LDL cholesterol. It has, however, to be highlighted that this study also included eight probands with 46,XY gonadal dysgenesis and results were not reported separately for the remaining CAIS probands. Furthermore, all probands received 1 mg norethisterone acetate in addition to 2 mg of 17b- estradiol orally. It is known that progestins can have independent effects on metabolism [31, 32] and similar effects should be expected for women with CAIS with an intact progesterone receptor (PR). But progestins usually result in a decrease in HDL-cholesterol in the general population. As women with CAIS to not have a uterus due to regression of the mullerian ducts, progestins are usually not indicated in these patients, as there is no evidence for additional health benefits and adverse effects probably prevail [33].

In addition, it has been demonstrated that there is a difference in the effects of estrogens on lipid levels depending on the application route [34]. While in postmenopausal women, both oral and transdermal application have beneficial effects on the HDL/LDL-cholesterol-ratios, this effect is more pronounced when taken orally [34, 35], while transdermal application is considered to have more favorable effects on triglyceride levels [35].

Finally, as expected, there were no significant differences in any other investigated parameters, including hemoglobin levels. Hemoglobin production is highly sensitive to testosterone [36] and its effects are also preserved on a female genetic background, as having been demonstrated by the fact that a few months of treatment in transmen are usually sufficient to increase its levels to those of the general male population [37]. That the effects of testosterone on hematopoiesis do not depend on aromatization [38] is in line with unchanged hemoglobin levels found in our study.

A strength of our study is that it is the first of its kind investigating two treatment options in a molecularly well-defined cohort of CAIS probands in a randomized controlled fashion. A limitation of our study is that the groups were rather small due to the relatively high drop-out rates and that power-analysis was not performed for secondary outcomes. This might have resulted in a bias. However, subjects who dropped out of the study did not differ in the investigate baseline characteristics in comparisons to those who successfully completed the study.

While the effects on cholesterol levels were quite strong, the study may be underpowered for the detection of more subtle group effects in other parameters. In addition, the subjects in our study were put on a fixed dosage of transdermal sex steroids. Although the corresponding dosage was selected on the basis of providing target concentrations according to the manufacturer’s average serum levels within the reference range of men and women from the general population, there is a high inter-individual variance due to differences in transdermal absorption. A dose titration scheme-based protocol might therefore have helped to average out these differences and higher estradiol levels than those achieved on average in our study population, may have further beneficial effects on bone structure [39, 40] and metabolic health surrogates [41]. In addition, anthropometric measures such as body composition and waist/hip circumference were not recorded, therefore we cannot determine if the observed changes in BMI were due to an increase in fat or lean mass or simple water retention.

Nevertheless, given the rarity of the disease and the paucity of studies on metabolic effects of hormone treatment in CAIS in general, in our opinion, this study adds valuable information to the understanding of the action of sex steroids on metabolism in this unique condition.

## Conclusion

In summary we could show both treatments seem to result in a worsened lipid profile while we did not detect major changes or group differences in other parameters. The exact mechanisms of this finding still must be determined. As we have shown before that testosterone might have beneficial effects in terms of sexual desire [5] in comparison to estradiol treatment in women with CAIS, its use may have additional value without any trade-off in terms of safety. There is evidence that women with CAIS have lower bone mineral density [10, 40] and this may be due to a combination of insufficient estradiol replacement as well as independent effects of androgen resistance, as androgens are know to have positive effects on bone [42]. However, it is unclear if any potential direct effect of testosterone on bone that is not mediated via aromatization to estradiol is of clinical relevance when adequate estradiol levels are achieved [43]. Further studies addressing this so far unanswered question would be appreciated.

## Data Availability

The datasets generated during and/or analyzed during the current study are not publicly available but are available from the corresponding author on reasonable request.
